# Structural and functional investigation of GajB protein in Gabija anti-phage defense

**DOI:** 10.1093/nar/gkad951

**Published:** 2023-10-28

**Authors:** Hyejin Oh, Jasung Koo, So Young An, Sung-Hyun Hong, Jeong-Yong Suh, Euiyoung Bae

**Affiliations:** Department of Agricultural Biotechnology, Seoul National University, Seoul 08826, Korea; Department of Agricultural Biotechnology, Seoul National University, Seoul 08826, Korea; Department of Agricultural Biotechnology, Seoul National University, Seoul 08826, Korea; Research Institute of Agriculture and Life Sciences, Seoul National University, Seoul 08826, Korea; Department of Agricultural Biotechnology, Seoul National University, Seoul 08826, Korea; Research Institute of Agriculture and Life Sciences, Seoul National University, Seoul 08826, Korea; Department of Agricultural Biotechnology, Seoul National University, Seoul 08826, Korea; Research Institute of Agriculture and Life Sciences, Seoul National University, Seoul 08826, Korea; Department of Agricultural Biotechnology, Seoul National University, Seoul 08826, Korea; Research Institute of Agriculture and Life Sciences, Seoul National University, Seoul 08826, Korea

## Abstract

Bacteriophages (phages) are viruses that infect bacteria and archaea. To fend off invading phages, the hosts have evolved a variety of anti-phage defense mechanisms. Gabija is one of the most abundant prokaryotic antiviral systems and consists of two proteins, GajA and GajB. GajA has been characterized experimentally as a sequence-specific DNA endonuclease. Although GajB was previously predicted to be a UvrD-like helicase, its function is unclear. Here, we report the results of structural and functional analyses of GajB. The crystal structure of GajB revealed a UvrD-like domain architecture, including two RecA-like core and two accessory subdomains. However, local structural elements that are important for the helicase function of UvrD are not conserved in GajB. In functional assays, GajB did not unwind or bind various types of DNA substrates. We demonstrated that GajB interacts with GajA to form a heterooctameric Gabija complex, but GajB did not exhibit helicase activity when bound to GajA. These results advance our understanding of the molecular mechanism underlying Gabija anti-phage defense and highlight the role of GajB as a component of a multi-subunit antiviral complex in bacteria.

## Graphical Abstract

**Graphical Abstract:**
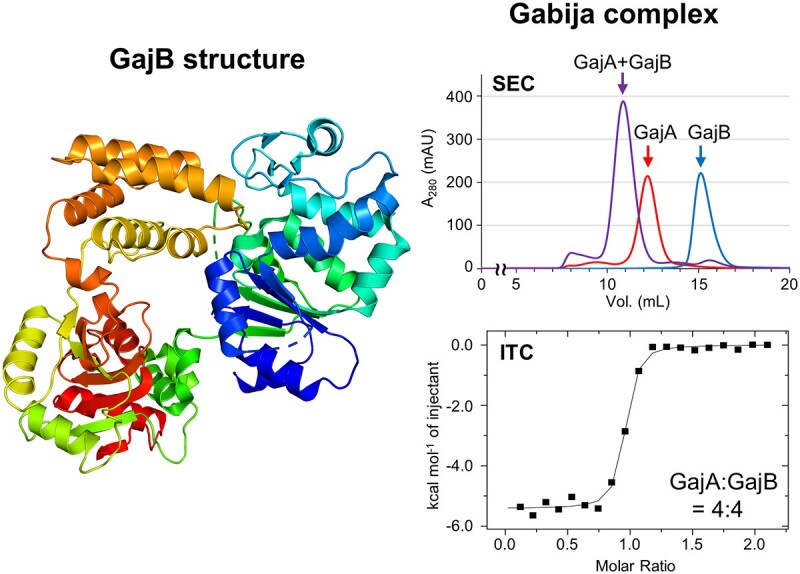

## Introduction

Bacteriophages, also called simply phages, are viruses that infect and kill bacteria and archaea (1). These microbial viruses are the most abundant biological entities in the biosphere and ubiquitously exist with their host (2,3). To fend off invading bacteriophages, bacteria and archaea have evolved a variety of anti-phage defense systems that function during distinct stages of infection (4,5). For some prokaryotic antiviral systems, defense mechanisms have been comprehensively investigated, including the restriction-modification and CRISPR-Cas systems (6,7). Molecular machineries employed by these anti-phage defense systems have been of particular interest since they have been repurposed for biotechnological applications such as recombinant DNA technique and genome editing (8–10). In recent years, systematic analyses of prokaryotic genomes and subsequent experimental validation have led to the identification of numerous anti-phage defense systems that had not been previously recognized (11,12). Few of these systems have been thoroughly characterized at molecular level (13–18).

The Gabija anti-phage defense system was first identified in *Bacillus cereus* and is present in >4000 prokaryotic genomes (11,19). After engineered to contain the Gabija system, bacteria could resist infections by multiple types of phages of *Siphoviridae* and *Podoviridae* (11). The Gabija system consists of two genes, *gajA* and *gajB*, both of which are indispensable for its anti-phage activity (11). The first component in the system, GajA, was originally predicted to be an ATP-dependent endonuclease of the overcoming lysogenization defect (OLD) family (11). The OLD proteins comprise a group of nucleases in bacteria, archaea and viruses, which contain P-loop ATPase and topoisomerase-primase (Toprim) domains (20–23). In OLD family proteins, the Toprim domain serves as a catalytic module that mediates metal-dependent cleavage of nucleic acid substrates (20,23). GajA was characterized experimentally as a sequence-specific DNA nicking endonuclease, whose active site is located in its Toprim domain (24). The endonuclease activity of GajA was regulated by NTP and dNTP concentrations, and nucleotide sensing was mediated by its ATPase-like domain (24).

The other component of the Gabija system, GajB, was predicted as a UvrD-like helicase (11), but its function is unknown. UvrD, known previously as helicase II in *Escherichia coli* (25), is a representative of superfamily 1 (SF1) helicases (26). This enzyme uses ATP hydrolysis for unwinding of dsDNA with 3′ to 5′ translocation polarity (27,28). The helicase function of UvrD is implicated in various cellular processes such as replication, recombination and repair (29–34). Other roles for UvrD have also been reported, including the translocation of ssDNA (35,36) and interaction with RNA polymerase (37).

In this study, we report the results of structural and functional analyses of GajB protein from the Gabija anti-phage defense system in *B. cereus* VD045. The crystal structure of GajB was determined and compared with that of UvrD helicase. Functional characterization for potential activities of GajB was performed, including helicase activity and DNA binding affinity. We demonstrated that GajB interacts strongly with GajA to form a stable heterooctameric complex. These findings highlight similarities and differences in structure and function between GajB and UvrD, suggesting a role for GajB as a component of a larger protein complex in Gabija-mediated bacterial anti-phage defense.

## Materials and methods

### Cloning, expression and purification

Synthetic *gajA* and *gajB* genes of *B. cereus* VD045 were ligated into pET21a with a C-terminal (His)_6_ tag and pET28a, which includes an N-terminal (His)_6_ -maltose binding protein (MBP) tag and a tobacco etch virus (TEV) protease cleavage site, respectively (Supplementary Figure S1). *E. coli* BL21(DE3) cells containing each individual construct were grown in lysogeny broth (LB) at 37°C until the optical density at 600 nm reached 0.7. Protein expression was induced with 0.5 mM isopropyl β-d-1-thiogalactopyranoside at 17°C for 16 h. The cells were harvested by centrifugation and resuspended in buffer [300 mM NaCl, 10% (w/v) glycerol, 5 mM β-mercaptoethanol, 30 mM imidazole, 20 mM tris(hydroxymethyl)aminomethane (Tris)–HCl pH 7.5]. After sonication and centrifugation, the supernatant was loaded onto a 5 mL HisTrap HP column (GE Healthcare, USA) pre-equilibrated with the same buffer. After washing, the bound proteins were eluted by applying a linear gradient of imidazole (up to 450 mM). The (His)_6_-MBP tag of GajB was cleaved by TEV protease and separated with the HisTrap HP column. The proteins were further purified by size exclusion chromatography (SEC) using a HiLoad 16/60 Superdex 200 column (GE Healthcare) equilibrated with buffer [300 mM NaCl, 5% (w/v) glycerol, 2 mM 1,4-dithiothreitol (DTT), 20 mM 4-(2-hydroxyethyl)-1-piperazineethanesulfonic acid (HEPES) pH 7.5].

For co-expression of the GajA:GajB complex, the synthetic *gajA* and *gajB* genes were ligated into pET28a, which includes an N-terminal (His)_6_-MBP tag and TEV protease cleavage site, and pET21a, which does not contain a tag, respectively (Supplementary Figure S1). *E. coli* BL21(DE3) cells were transformed with these constructs. Protein expression and purification of the (His)_6_-MBP-GajA:GajB complex were performed as described for the individual Gabija proteins. The (His)_6_-MBP tag of GajA was removed prior to the final SEC as described above.

### Crystallization and structure determination of GajB

To determine the crystal structure of GajB using single-wavelength anomalous diffraction, selenomethionyl protein was expressed in *E. coli* BL21(DE3) cells grown in M9 medium supplemented with selenomethionine, as described previously (38). The selenomethionyl GajB was purified in the same manner as described above for the native GajB protein. The GajB crystals were obtained at 20°C by the hanging-drop vapor diffusion method from 175 μM protein solution in buffer [300 mM NaCl, 5 mM DTT, 5% (w/v) glycerol, 20 mM HEPES pH 7.5] mixed with an equal amount of reservoir solution [20% (v/v) polyethylene glycol 400, 20% (w/v) ethylene glycol, 0.1 M Tris–HCl pH 8.5]. The crystals were flash-frozen in liquid nitrogen. Diffraction data were collected at the beamline 7A of the Pohang Accelerator Laboratory at 100 K. Diffraction images were processed with HKL2000 (39). Determination of selenium positions, density modification and initial model building were performed using PHENIX (40). The structure was completed using alternate cycles of manual fitting in Coot (41), and refinement in PHENIX (40). The stereochemical quality of the final model was evaluated using MolProbity (42).

### Helicase activity assay

Helicase activity assay was performed as described previously (43) with modifications. DNA substrates were generated by annealing a 25-mer oligonucleotide with a fluorescein label to 40- or 25-mer oligonucleotides to form tailed or blunt-ended dsDNAs. Then, 0.75 μM DNA substrate was incubated with proteins (3.75 μM each) in 12 μl of buffer [5 mM MgCl_2_, 1 mM DTT, 0.75 μM bovine serum albumin (BSA), 20 mM Tris–HCl pH 7.5] at 25°C for 10 min. The reaction was initiated by adding 8 μl of start solution including 12.5 mM ATP and 22.5 μM trapping oligonucleotide to prevent re-annealing. This trapping oligonucleotide is complementary to the fluorescein-labeled strand of the substrate and different in size from the original complementary strand. The reactions were incubated at 25°C for 30 min and stopped by the addition of 5 μl of proteinase K (173 μM) and 5 μl of quenching solution [0.5 M EDTA, 10% (w/v) sodium dodecyl sulfate (SDS), 25% (w/v) glycerol, 0.1% (w/v) bromophenol blue]. The reaction products were analyzed by electrophoresis on a 20% native polyacrylamide gel. The DNA oligonucleotides used in the assay were commercially synthesized and purified by high-performance liquid chromatography (Macrogen, Korea). Their sequences are listed in Table S1. These DNAs do not contain the recognition sequence of the GajA endonuclease. As a positive control for the helicase activity assay, we used *E. coli* UvrD (44). The gene encoding UvrD was amplified using polymerase chain reaction from genomic DNA of *E. coli* DH5α. The cloning, expression and purification of *E. coli* UvrD were performed as described above for GajB.

### DNA binding assay

DNA binding of proteins was tested by electrophoretic mobility shift assay (EMSA). The 3′-tailed dsDNAs (5 μM) were incubated with proteins (10 μM) in 20 μl of buffer (5 mM MgCl_2_, 1 mM DTT, 0.75 μM BSA, 20 mM Tris–HCl pH 7.5) at 25°C for 90 min. After the addition of 4 μl of loading buffer [250 mM NaCl, 25 mM MgCl_2_, 5 mM DTT, 60% (w/v) glycerol, 0.1% (w/v) bromophenol blue, 100 mM Tris–HCl pH 7.5], the samples were analyzed on a 2% agarose gel and visualized by ethidium bromide staining.

### ATPase activity assay

ATPase activity was measured by using the PiColorLock™ phosphate assay kit (Abcam, UK). Proteins (5 μM each) were incubated with 0.5 mM ATP at 30°C for 15 min in buffer (10 mM MgCl_2_, 1 mM DTT, 20 mM Tris–HCl pH 7.0). The reactions were stopped by the addition of the PiColorLock™ reagent containing malachite green dye, which undergoes an absorance change at 635 nm in the presence of inorganic phosphate. Concentrations of released inorganic phosphates were determined by using phosphate standard solutions.

### DNA translocase activity assay

DNA translocase activity was measured by using triplex displacement assay as previously described (45,46). To generate the triplex DNAs, 5′ fluorescein-labeled 22-mer triplex forming oligonucleotide (TFO) was hybridized to 151 bp linear DNA at 57°C for 15 min in buffer (12.5 mM MgCl_2,_ 10 mM MES pH 5.5) and cooled down to 4°C for overnight. Proteins (0.5–10 μM) were mixed with the triplex DNAs (0.2 μM) in 12.8 μl of buffer (10 mM MgCl_2_, 1 mM DTT, 50 mM Tris–HCl pH 7.5) and 3.2 μl of 10 mM ATPs were then added to start the reactions. After incubation at 25°C for 10 min, the reactions were stopped by the addition of 4 μl GSM buffer [15% (w/v) glucose, 3% (w/v) SDS, 250 mM 3-(morpholin-4-yl)propane-1-sulfonic acid (MOPS) pH 5.5). The samples were analyzed on a 7% tris-acetate polyacrylamide gel.

### Nuclease activity assay

Nuclease activity assays were performed as previously described (24). DNA substrates were generated by annealing two complementary 56-mer oligonucleotides. Their sequences are listed in Table S1. DNA substrates (1 μM) were incubated with proteins (5 μM each) in 20 μl of buffer (1 mM MgCl_2_, 0.75 μM BSA, 20 mM Tris–HCl pH 9.0) at 37°C for 20 min. The reactions were stopped by the addition of 4 μl of quenching buffer [20 mM EDTA, 60% (w/v) glycerol, 0.1% (w/v) bromophenol blue, 120 mM Tris–HCl pH 9.0] and 5 μl of proteinase K (173 μM). The reaction products were analyzed on a 20% native polyacrylamide gel and visualized by ethidium bromide staining.

### Analytical SEC

Analytical SEC was performed using Superdex 200 10/300 GL column (GE Healthcare) pre-equilibrated with buffer [300 mM NaCl, 2 mM DTT, 5% (w/v) glycerol, 20 mM HEPES pH 7.5]. Samples (20 μM each) were incubated at 4°C for 1 h and loaded onto the column at a flow rate of 0.5 ml/min. Elution fractions were analyzed by SDS-polyacrylamide gel electrophoresis (PAGE) and visualized by Coomassie staining.

### SEC-multi-angle light scattering (MALS)

SEC-MALS analyses were performed using Superdex 200 Increase 10/300 GL column (GE Healthcare) coupled to the miniDAWN (3-angle)/Optilab instrument (Wyatt Technology). The column was pre-equilibrated with buffer [300 mM NaCl, 2 mM DTT, 5% (w/v) glycerol, 20 mM HEPES pH 7.5]. Samples (20 μM each) were loaded onto the column at a flow rate of 0.8 mL/min at 25ºC. Data were analyzed using ASTRA software (Wyatt Technology).

### Isothermal titration calorimetry (ITC)

ITC was performed at 4°C using a MicroCal iTC200 Calorimeter (Malvern). 260 μl of 20 μM GajB in buffer [300 mM NaCl, 1 mM Tris(2-carboxyethyl)-phosphine, 10% (w/v) glycerol, 20 mM HEPES pH 7.5] was titrated with 20 consecutive 2-μl injections of 200 μM GajA. Data processing and analysis were conducted using Origin software (OriginLab).

## Results

### Crystal structure of GajB

The Gabija anti-phage defense system includes two protein components, GajA (67.0 kDa) and GajB (57.1 kDa) (11). GajA was previously characterized as a sequence-specific DNA endonuclease (24), whereas the function of GajB is unclear. To gain structural insight into the function of GajB in Gabija-mediated anti-phage defense, the crystal structure of selenomethionyl GajB was determined to a resolution of 2.0 Å using single-wavelength anomalous diffraction. Data collection, phasing and refinement statistics are presented in Table 1. The asymmetric unit contained a single GajB chain and 265 water molecules. Several GajB residues (1, 18–26, 62–66 and 193–198) were not modeled in the final structure due to insufficient electron density.

**Table 1.** Data collection, phasing and refinement statistics^a^

Space group	*P*2_1_2_1_2_1_
Unit cell parameters (Å)	*a* = 68.66, *b* = 70.26, *c* = 145.76, α=β=γ=90°
Solvent content (%)	61.0
Wavelength (Å)	0.9793
**Data collection statistics**	
Resolution range (Å)	50.00–2.00 (2.70–2.00)
Number of reflections	48212 (4743)
Completeness (%)	99.7 (100.0)
*R*_merge_^b^	0.106 (1.027)
CC1/2	0.990 (0.893)
CC*	0.998 (0.971)
Redundancy	14.3 (14.6)
Mean I/σ	16.1 (3.0)
**Phasing statistics**	
f′, f″ used in phasing	–7.76, 5.28
Figure of merit	0.39
**Refinement statistics**	
Resolution range (Å)	50.00–2.00 (2.70–2.00)
*R*_cryst_^c^/*R*_free_^d^ (%)	19.7/23.4
RMSD bonds (Å)	0.008
RMSD angles (°)	0.95
Average B-factor (Å^2^)	46.1
Number of water molecules	265
Ramachandran favored (%)	98.5
Ramachandran allowed (%)	1.5

^a^Values in parentheses are for the highest-resolution shell.

^b^ *R*_merge_ =Σ_*h*_Σ|*I_i_*(*h*) – <*I*(*h*)> |/Σ_*h*_Σ_*i*_*I_i_*(*h*), where *I_i_*(*h*) is the intensity of an individual measurement of the reflection and <*I*(*h*)> is the mean intensity of the reflection.

^c^ *R*_cryst_ = Σ_h_||*F*_obs_| – |*F*_calc_||/Σ_h_|*F*_obs_|, where *F*_obs_ and *F*_calc_ are the observed and calculated structure factor amplitudes, respectively.

^d^ *R*_free_ was calculated as *R*_cryst_ using ∼5% of the randomly selected unique reflections that were omitted from structure refinement.

**Figure 1.**
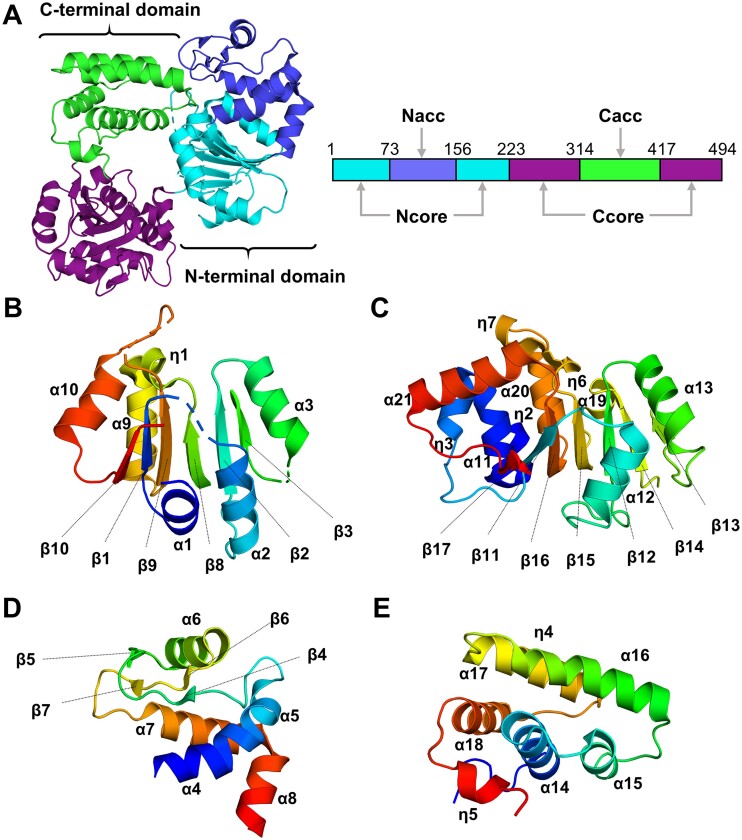
Crystal structure of GajB reveals a four-subdomain architecture. (**A**) Overall structure of GajB. Ncore, Nacc, Ccore and Cacc subdomains of GajB are in cyan, blue, purple and green, respectively. Schematic linear representation of GajB subdomain organization is also shown with residue numbers indicating the subdomain boundaries. (B–E) Structures of the Ncore (**B**), Ccore (**C**), Nacc (**D**) and Cacc (**E**) subdomains of GajB. Each subdomain is shown in rainbow format from the N terminus (blue) to the C terminus (red). Secondary structural elements are also indicated.

The crystal structure of GajB revealed a four-subdomain architecture, in which N- and C-terminal domains are each divided further into a RecA-like core subdomain and an accessory subdomain (Figure 1A). The two core subdomains share the common structural feature of the RecA fold, in which a central β-sheet is surrounded by multiple helical elements (47,48). The N-terminal core (Ncore) subdomain (residues 1–72 and 156–222) contains a six-stranded parallel β-sheet (β10–β1–β9–β8–β2–β3) sandwiched between two helical layers (α1–α3 and α9, α10, η1) (Figure 1B). The C-terminal core (Ccore) subdomain (residues 223–313 and 417–494) possesses a seven-stranded parallel β-sheet (β17–β11–β16–β15–β12–β14–β13) with four α- and four 3_10_-helices (α11, α19, α20, α21 and η2, η3, η6, η7) on one side of the central β-sheet and two α-helices (α12, α13) on the other side (Figure 1C).

The two accessory subdomains are formed as inserts within the respective core subdomains (Figure 1A). The N-terminal accessory (Nacc) subdomain (residues 73–155), which joins between β3 and β8 of the Ncore subdomain, is composed of five α-helices (α4–α8) and two parallel β-sheets (β4–β6 and β5–β7) (Figure 1D). Four α-helices (α4, α5, α7 and α8) pack against α9 and β3 of the Ncore subdomain. An additional α-helix (α6) and the two small β-sheets are found adjacent to α7 of the four helical assembly. In the C-terminal accessory (Cacc) subdomain (residues 314–416), which is inserted between β13 and β14 of the Ccore subdomain, five α-helices (α14–α18) and two 3_10_-helices (η4, η5) comprise a compact three-layered globular structure (Figure 1E). Three α-helices (α14, α15, α18) are aligned on the top of a short 3_10_-helix (η5) and connecting loops. Two longer α-helices (α16, α17) and a 3_10_-helix (η4) are stacked on the three aligned α-helices in an approximately perpendicular orientation.

### Structural comparison of GajB and UvrD helicase

**Figure 2.**
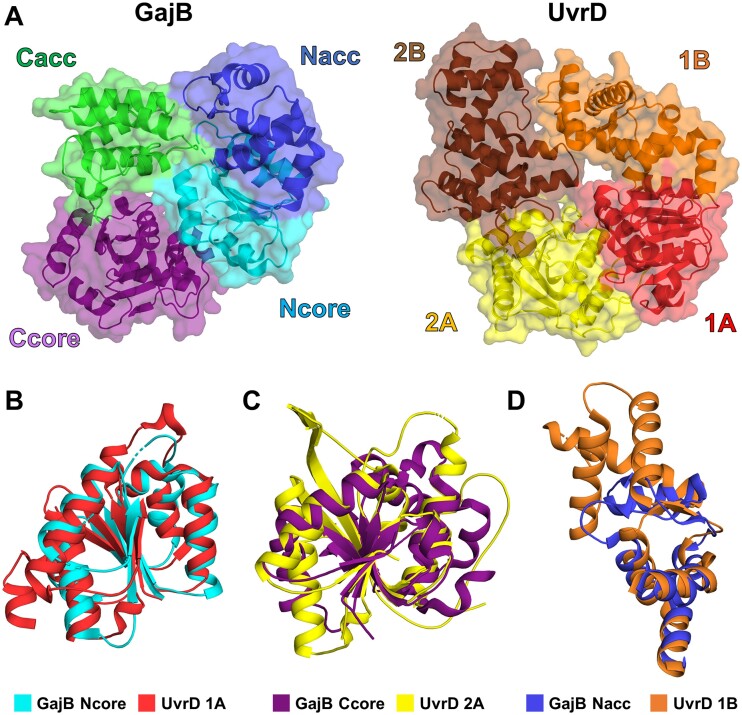
Structural similarity between GajB and UvrD. (**A**) Side-by-side comparison of GajB and UvrD (PDB ID: 3LFU) structures. GajB and UvrD share a four-subdomain architecture. Subdomains of GajB are colored as in Figure 1. The 1A, 1B, 2A and 2B subdomains of UvrD are shown in red, orange, yellow and brown, respectively. (B–D) Structural alignments of subdomains between GajB and UvrD. The Ncore (**B**) and Ccore (**C**) subdomains of GajB are superposed well with the 1A and 2A subdomains of UvrD, respectively. Structural alignment of the Nacc (**D**) subdomain in GajB with the 1B subdomain of UvrD reveals only a partial similarity. Structural superposition is not shown for the Cacc subdomain of GajB and 2B subdomain of UvrD because there was no significant match between them.

GajB was originally predicted to be a UvrD-like helicase (11), and a BLAST search (49) identified SF1 DNA helicases as the closest homologues of GajB (Table S2). The crystal structure of GajB also displayed a considerable similarity with that of *E. coli* UvrD (Figures 2A and S2). A search using Dali server (50) identified several SF1 helicases as structural neighbors of GajB, among which the apo form of *E. coli* UvrD (PDB ID: 3LFU) exhibited the highest structural resemblance (Table S3). GajB shares the four-subdomain architecture with UvrD that includes two RecA-like subdomains (1A and 2A) and two additional subdomains (1B and 2B) (Figure 2A) (28). The most significant match between GajB and UvrD was for the two RecA-like subdomains (Figure 2B, C). The root-mean-square deviation (RMSD) values between the Ncore and Ccore subdomains of GajB and their counterparts (the 1A and 2A subdomains, respectively) in UvrD were 1.9 Å and 2.8 Å for the 115 and 141 Cα atoms, respectively. Their Dali Z-scores were 15.5 and 12.7, respectively.

Structural differences were noted in the other subdomains. When the Nacc subdomain of GajB was superposed with the 1B subdomain of UvrD, only partial structural similarity was observed (Figure 2D). The *Z*-score was 4.8 and the RMSD value was 2.5 Å for 68 Cα atoms. Although the four α-helices (α4, α5, α7, α8) and one of the two β-sheets (β4–β6) in GajB were well-aligned to the corresponding portion of UvrD, a mismatch was evident for the rest of the Nacc subdomain, including the α6-helix and the other β-sheet (β5–β7) (Figure 2D). In the Cacc subdomain of GajB, it is difficult to recognize even a partial structural resemblance to the 2B subdomain of UvrD. When these subdomains were structurally aligned, the *Z*-score was only 4.3, and the RMSD value was calculated to be 3.7 Å for 90 Cα atoms.

**Figure 3.**
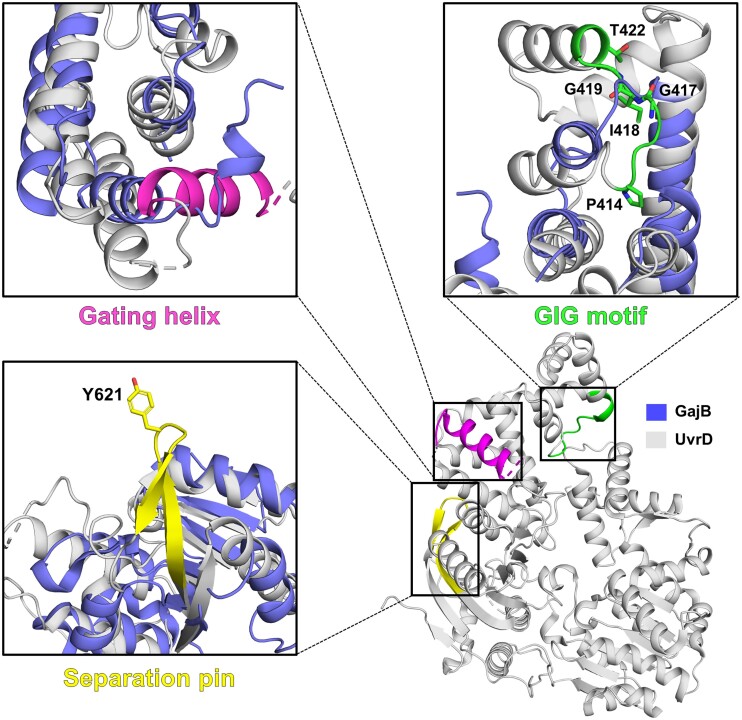
GajB lacks functionally important structural elements of UvrD. The gating helix (magenta), GIG motif (green) and separation pin (yellow) are structural elements that are important for helicase activity of UvrD. These three elements are highlighted in the structure of UvrD (PDB ID: 3LFU; grey). In close-up views, the corresponding parts in the GajB structure (blue) are superposed for comparison.

Notably, several structural elements in UvrD, which are important for its helicase function are not conserved in GajB (Figures 3 and S2). The gating helix and the GIG motif in subdomain 2B of UvrD, which are involved in formation of the ssDNA gateway and dsDNA binding, respectively (28), are missing in GajB. It also lacks the separation pin, which is a β-hairpin motif in the 2A subdomain of UvrD that contacts the ds-ssDNA junction (28). Mutation(s) in these regions disrupted the DNA unwinding function of UvrD (28).

### Functional investigation for potential activities of GajB

**Figure 4.**
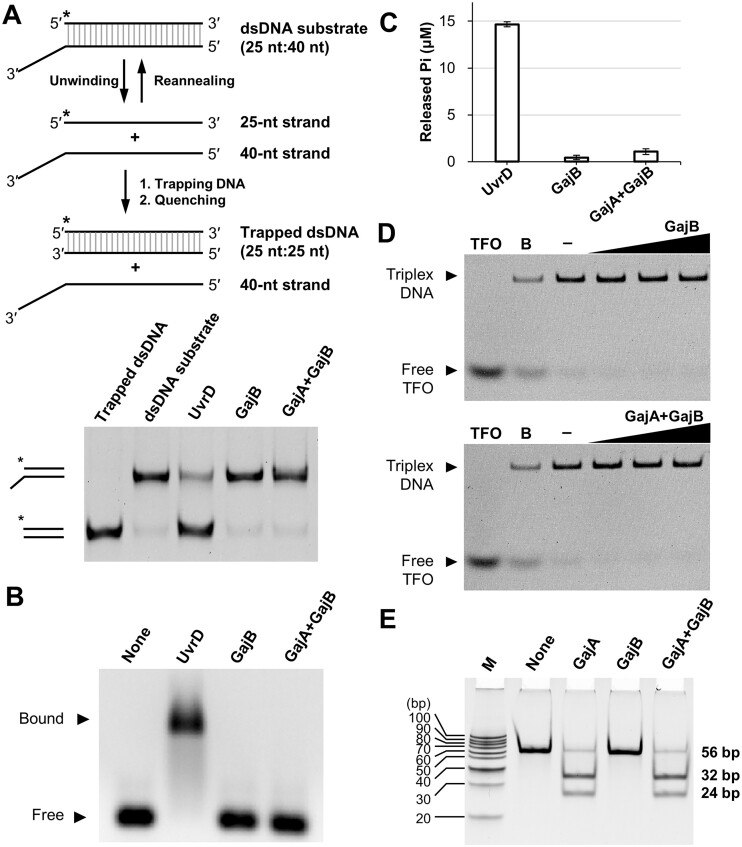
Activity assays of Gabija proteins. (**A**) Helicase activity assay of GajB and GajA:GajB complex. 3′-Tailed dsDNA substrates were incubated with proteins and analyzed by PAGE. Asterisks indicate fluorescein labels. (**B**) DNA binding assay of GajB and GajA:GajB complex. Agarose-based EMSAs of proteins (10.0 μM each) were conducted with 3′-tailed dsDNA substrate. (**C**) ATPase activity assay of GajB and GajA:GajB complex. Amounts of released inorganic phosphate from three independent experiments are shown as mean ± standard error of the mean (SEM). (**D**) DNA translocase activity assays of GajB and GajA:GajB complex. DNA translocation was analyzed by triplex displacement assay, in which fluorescein-labeled triplex forming oligonucleotide (TFO) was hybridized to linear dsDNA, and translocation was measured after the addition of ATP and proteins (0.5, 2.5 and 10.0 μM each). Samples were analyzed by PAGE. ‘B’ represents boiled sample. (**E**) Nuclease activity assay of GajA and GajA:GajB complex. Proteins were incubated with DNA substrate containing GajA recognition sequence. Reaction products were analyzed by PAGE and visualized by ethidium bromide. The sequences of oligonucleotides used in the assays are listed in Table S1. Uncropped gel images are shown in Figure S8.

Because our structural analyses suggest that GajB may not share all the functional characteristics of UvrD as a DNA helicase, we experimentally tested the helicase function of GajB. In helicase activity assays, we introduced trapping DNA oligonucleotides to prevent spontaneous re-annealing of the separated ssDNAs (43). The trapping DNA is complementary to the fluorescein-labeled strand of the dsDNA substrates, and its size is distinct from that of the original complementary strand, allowing the separation and detection of helicase-catalyzed DNA products (Figure 4A).

We first tested the helicase function of GajB with the 3′-tailed DNA duplex because most SF1 helicases, including UvrD, preferentially unwind this type of dsDNA substrate (44,51). In this experiment, we did not detect helicase activity of GajB (Figure 4A), whereas the positive control, *E. coli* UvrD, exhibited robust DNA-unwinding activity. We also performed additional assays, in which the fluorescein was labeled at the opposite end of the same dsDNA substrate. The results from these assays were essentially identical to those obtained with the original experimental setting (Supplementary Figure S3A), indicating that the location of the fluorescein label did not matter. Next, we questioned whether GajB could unwind other types of dsDNAs, but no helicase activity was not observed for 5′-tailed and blunt-ended substrates (Supplementary Figure S3B, C). We also examined the binding of GajB to the 3′-tailed DNA duplex without ATP since such DNA binding affinity is documented for SF1 helicases including UvrD (28,52,53). However, no binding was detected for GajB, but *E. coli* UvrD bound the DNA substrate in the same experimental setting (Figure 4B). Consistent with these results, the electrostatic potential surface of GajB is considerably different from that of UvrD, including its positively charged DNA binding interface (Supplementary Figure S4). These findings suggest that GajB may not be a stand-alone helicase enzyme.

We then explored possibilities that GajB may possess other enzymatic activities. We examined whether GajB could perform ATP hydrolysis. However, no such activity was detected for GajB (Figure 4C), while UvrD hydrolyzed ATP as previously reported (54). We also tested DNA translocase activity for GajB since some bacterial antiphage defense proteins such as the type I restriction endonuclease EcoR124I couple ATP hydrolysis to DNA translocation (45,46). In our assay using a fluorescent DNA triplex (Figure 4D), GajB did not exhibit DNA translocase activity. Finally, we tested whether GajB could influence the function of GajA, which was previously demonstrated as a sequence-specific DNA endonuclease (24). In our experiments, GajA could cleave DNA substrates with and without GajB (Figure 4E), suggesting that GajB has no effect on the nuclease activity of GajA.

### GajB interacts with GajA to form a stable Gabija protein complex

**Figure 5.**
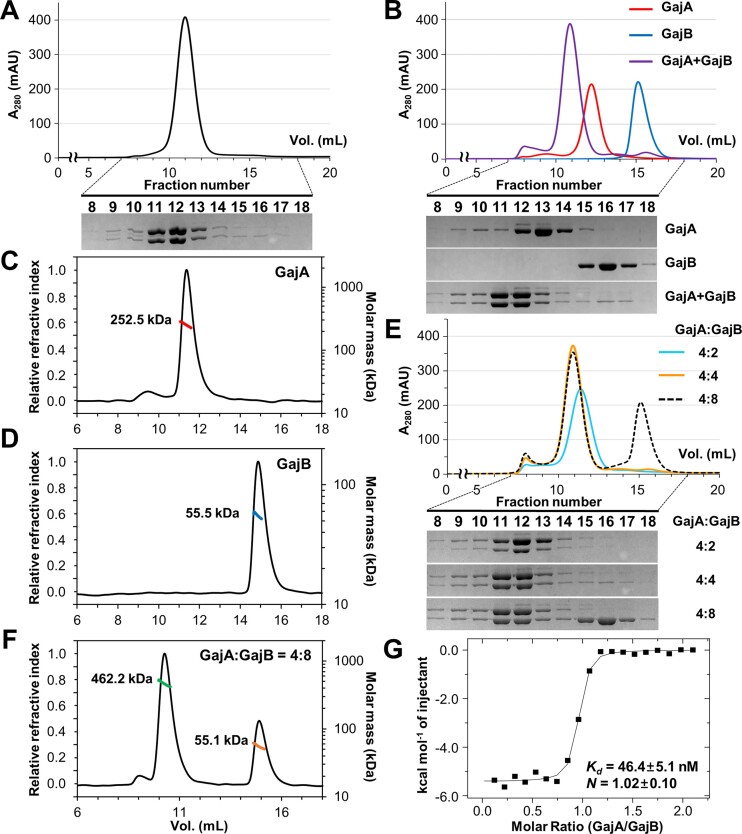
GajB interacts with GajA to form a 4:4 Gabija complex. (**A**) Co-elution of co-expressed GajA and GajB in SEC. When GajA and GajB were expressed together in *E. coli*, they co-purified in SEC. (**B**) Formation of Gabija complex from individually purified GajA and GajB proteins in SEC. When incubated together prior to injection, individually purified GajA and GajB proteins migrated together (purple) during SEC with a smaller retention volume than those for GajA (red) and GajB (blue). (C, D) SEC-MALS analyses of GajA and GajB. Oligomeric states of GajA (**C**) and GajB (**D**) were investigated by SEC-MALS. The average molar masses corresponding the SEC peaks are consistent with tetrameric GajA and monomeric GajB. Theoretical molecular weights of the GajA tetramer and the GajB monomer used in these experiments are 272.2 and 57.3 kDa, respectively. (**E**) SEC analysis of the stoichiometry of Gabija complex. GajA was pre-incubated with increasing amounts of GajB, and the mixtures were analyzed by SEC. Until the molar ratio of GajA to GajB reached 4:4 (cyan and orange lines), the retention volume decreased and the peak height of the complex increased. Addition of extra GajB (black dashed line) did not change the peak of the complex, but resulted in emergence of an unbound GajB peak. (**F**) SEC-MALS analysis of Gabija complex. GajA was pre-incubated with an excess amount of GajB, and the mixture was analyzed by SEC-MALS. The molar ratio of GajA to GajB was 4:8. The average molar masses corresponding the two SEC peaks were consistent with the 4:4 GajA:GajB complex and monomeric GajB. The theoretical molecular weight of the 4:4 GajA:GajB complex used in this experiment is 501.3 kDa (**G**) ITC trace for GajA binding to GajB. The isotherm is representative of triplicate measurements. The dissociation constant (*Kd*) and binding ratio (*N*) are presented as mean ± SEM. Data for the three independent ITC experiments are shown in Figure S6. Uncropped gel images are shown in Figure S8.

The helicase activities of some SF1 enzymes are poor *in vitro*, and in some cases their activities are greatly enhanced by interactions with accessory proteins (33,55–61). Thus, we explored the possibility that GajB interacts with another protein to form a larger defense complex, as in other bacterial anti-phage or anti-plasmid immune systems such as the CRISPR-Cas (62–65), Wadjet (66,67), RADAR (15,16), Avs (17) and DISARM (68) systems. Since the Gabija system is composed of two proteins, GajA and GajB (11), we first analyzed the formation of a GajA:GajB complex. When (His)_6_-MBP tagged GajA was co-expressed with untagged GajB in *E. coli*, the two proteins co-purified after cleavage of the (His)_6_-MBP tag and co-eluted in SEC (Figure 5A), indicating the formation of a stable GajA:GajB complex. To verify the association of the two Gabija proteins, we performed SEC analyses with individually purified GajA and GajB proteins. After pre-incubation, the separately purified GajA and GajB migrated together as a larger single peak in SEC with a shorter retention time than those for the two individual proteins alone (Figure 5B), suggesting that the two proteins directly interact to form a two-component complex.

Next, we examined the stoichiometry of GajA:GajB complex formation. By SEC-MALS and analytical SEC, GajA and GajB were estimated to be tetrameric and monomeric in solution, respectively (Figures 5C, D and S5). We next performed additional analytical SEC runs, in which GajA was pre-incubated with increasing amounts of GajB. In these experiments, we observed a decrease in retention time with an increase in peak height for the Gabija complex until the molar ratio of GajA to GajB reached 4:4 (cyan and orange lines in Figure 5E). When extra GajB was added, the peak of the complex was unchanged and one corresponding to unbound GajB appeared (black dashed line in Figure 5E). These results suggest that a maximum of four GajB monomers can bind to a single GajA tetramer. The SEC-MALS analysis of the GajA:GajB complex also supported the 4:4 molar ratio in the full assembly (Figure 5F).

For quantitative analysis of complex formation, we performed ITC, in which GajB was titrated with GajA (Figures 5G and S6). In the ITC experiments, the dissociation constant (*K*_d_) was determined to be ∼46.4 nM, indicating the formation of a stable protein complex. The molar binding ratio (*N*) of GajA to GajB was estimated to be ∼1.0, which is consistent with the results of the analytical SEC and SEC-MALS experiments. Taken together, our analyses demonstrated that the GajA and GajB proteins form a stable complex composed of a single GajA tetramer and four GajB monomers. The repeated observations of the 4:4 molar ratio in different experimental settings suggest that the heterooctameric configuration of the Gabija complex is biologically relevant, and GajB—as a component of a larger protein complex—plays a role in bacterial anti-phage defense.

Once the formation of the GajA:GajB complex was experimentally confirmed, we tested whether the interaction with GajA activated the helicase function of GajB. Again, for several SF1 enzymes, helicase activity required interactions with other protein factors (33,55–61). In helicase activity assays, the GajA:GajB complex did not unwind any of the dsDNA substrates that possessed a 3′-ssDNA tail, a 5′-ssDNA tail, or a blunt-end (Figures 4A and S3), indicating that interaction with GajA did not activate the helicase function of GajB. We also tested whether the GajA:GajB complex could bind DNAs without ATP as observed in many UvrD-like helicases, but no binding was detected (Figure 4B). The Gabija complex did not display ATPase or DNA translocase function either (Figure 4C, D). Therefore, GajB did not exhibit the tested enzymatic activities alone or in complex with GajA, suggesting it to have other function(s) for anti-phage defense.

## Discussion

GajB was previously proposed as a UvrD-like DNA helicase based on sequence analysis (11). However, our results suggest that GajB may not function as a helicase enzyme. In DNA unwinding assays for GajB and its complex with GajA, helicase activity was not detected with various types of dsDNA substrates (3′-tailed, 5′-tailed and blunt-ended) (Figures 4A and S3). Moreover, GajB did not bind to DNAs in EMSA experiments (Figure 4B). Our structural analyses of GajB also support the possibility that GajB does not possess DNA helicase activity. Although the crystal structure of GajB revealed high overall similarity to that of *E. coli* UvrD, critical local differences were recognized between the two structures. We found that several functionally important structural elements of UvrD are not present in GajB, such as the gating helix, the GIG motif and the separation pin (Figure 3).

In the crystal structure of GajB, the Nacc and Cacc subdomains revealed unique structural features distinct from UvrD. Thus, we thought that the identification of their structural neighbors with known function(s) would provide insight into to the biological role of GajB. However, searches using the Dali server (50) did not yield meaningful results. The top three structural neighbors of the Nacc subdomain were UvrD-like helicases, and the other retrieved structures exhibited only partial similarities with limited numbers of superimposable residues (Table S4). A search for the Cacc subdomain of GajB did not return a significant match. The structural neighbors were identified with low *Z*-scores and high RMSD values (Table S5), indicating that the structural similarities are not enough to predict the function of GajB.

Cheng *et al.* recently reported functional analyses of GajB (69). They found that the Gabija gene cassette encodes two colinear GajB proteins (69). The longer form contains five additional N-terminal residues compared to the shorter GajB, which was originally reported by Doron *et al.* (11). This long-form GajB exhibited robust ATP hydrolysis activity but no helicase activity (69). Based on structural analyses, we suspect that the ATPase activity of the long-form GajB results from its strong binding to ATP. The structural alignment of GajB and UvrD revealed extra residues at the N-terminus of UvrD (Supplementary Figure S7). In the AMPPNP-bound UvrD structure (PDB ID: 2IS4), these additional N-terminal residues formed a short helix and a connecting loop, which is involved in the interaction with the adenine ring of the bound ATP analogue (Supplementary Figure S7). Indeed, our attempts to crystallize GajB with ATP, ADP and AMPPNP were unsuccessful. This is likely due to the lack of the N-terminal structural elements, which are important for binding to the adenine base of the ligands. It is noteworthy that most of other active site residues for the ATPase function are conserved in GajB, including those contacting the phosphate and ribose groups of ATP (Supplementary Figure S7).

We demonstrated that GajB forms a 4:4 complex with GajA, the other protein component of the Gabija defense system. Other researchers have reported the formation of the two-component Gabija complex (69,70). Cheng *et al.* observed the formation of the GajA:GajB complex *in vitro* and *in vivo* (69). Antine and coworkers determined the crystal structure of the Gabija complex, in which two sets of GajB dimers bind to opposite sides of a GajA tetramer (70). Mutations in the GajA–GajB hetero-oligomerization interface markedly reduced antiviral activity (70), highlighting the importance of complex formation in the Gabija anti-phage defense.

Despite the recent progress in characterization of the Gabija system, it is unclear whether GajB possesses helicase activity. Consistent with our inability to detect UvrD-like helicase activity for GajB (Figure 4A), Cheng *et al.* reported that long-form GajB did not unwind dsDNAs (69). Because activation of SF1 helicases is coupled to the rotation of the 2B subdomain and often requires self-assembly or interaction with accessory factors (28,71–73), we tested the helicase activity of the GajA:GajB complex, but none was detected (Figures 4A and S3). In the Gabija complex, the GajB protomers exhibit only partial rotation of the 2B subdomain (70). This intermediate position was also observed in our monomeric GajB structure (Figure 1A), indicating that dimerization and interaction with GajA may not be sufficient to induce a conformational change to the fully rotated active state. In the complex structure, the GajB dimerization interface is minimal, and mutations in this interface had no effect on phage resistance (70).

Although both GajA and GajB are essential for antiviral defense *in vivo* (11,69), GajA alone is sufficient to degrade target DNAs *in vitro* (69,70), suggesting roles for GajB in other stages of Gabija anti-phage defense. GajB may be involved in recruiting unknown host factor(s) during *in vivo* antiviral defense. In CRISPR-mediated immunity, host factors other than Cas proteins, such as RNase III and Integration Host Factor, are crucial components of the defense mechanism (74,75). It is also possible that GajB participates in sensing of phage activities. Cheng and coworkers suggested a model for Gabija anti-phage defense, in which GajB recognizes phage invasion by detecting DNA termini generated by phage DNA replication and recombination (69). Further work is needed to evaluate the molecular mechanism of Gabija anti-phage defense, including the role of GajB protein.

## Supplementary Material

gkad951_supplemental_file

## Data Availability

The atomic coordinates and structure factors are available in the Protein Data Bank at https://www.rcsb.org/ under the accession number 8IUD.
