# Supplementary material for: Structural and functional investigation of GajB protein in Gabija anti-phage defense
Source: Nucleic Acids Res. 2023 Oct 28;51(21):11941–51. doi: 10.1093/nar/gkad951 (PMC10681800; doi:10.1093/nar/gkad951)
Supplement: gkad951_supplemental_file [file gkad951_supplemental_file.pdf]

Supplementary Data

Table S1. DNA oligonucleotides used in this study

Type	Size (nt)	5′ to 3′ sequence
Forward strand	25	TCGGTACGACCTGCGAGCACTGCTT
Reverse strand for 3′ tail	40	AAGCAGTGCTCGCAGGTCGTACCGATTTTTTTTTTTTTTT
Reverse strand for 5′ tail	40	TTTTTTTTTTTTTTTAAGCAGTGCTCGCAGGTCGTACCGA
Reverse strand for blunt end	25	AAGCAGTGCTCGCAGGTCGTACCGA
Forward primer for triplex displacement assay	45	AAGAAAAGAAAGAAGAAAGAAAGTATGTTGTGTGGAAT TGTGAGC
Reverse primer for triplex displacement assay	20	CACGACGTTGTAAAACGACG
Triplex forming oligonucleotide (TFO)	22	TTCTTTTCTTTCTTCTTTCTTT
Forward strand for nuclease activity assay	65	TTTTTTTTTTTTTTTTTAATAACCCGGTTATTTTTTTTTTTT TTTTTTTTTTTTTT
Reverse strand for nuclease activity assay	65	AAAAAAAAAAAAAAAAAAAAAAAAAATAACCGGGTTAT TAAAAAAAAAAAAAAAAA

Table S2. Top ten protein sequences most similar to GajB in BLAST (1)

Protein	Organism	Total score	Coverage	E-value	% id	Length	Accession number
DNA helicase II	*Bacillus* sp. AFS015896	986	100	0	98.38	494	PFA61565.1
ATP-dependent helicase	*Priestia* sp. TSO9	596	99	0	59.84	501	WP_230932872.1
ATP-dependent helicase	*Clostridium tunisiense*	503	98	1.00E-171	53.86	495	WP_017413921.1
ATP-dependent helicase	*Intestinibacter bartlettii*	478	98	8.00E-162	51.41	502	WP_007285568.1
ATP-dependent helicase	*Neobacillus mesonae*	491	99	7.00E-167	51.00	495	WP_127484831.1
ATP-dependent helicase	*Alkalibacillus almallahensis*	497	98	3.00E-169	50.10	493	WP_167261860.1
AAA family ATPase	*Virgibacillus halodenitrificans*	486	99	6.00E-165	49.70	493	MYL56603.1
ATP-dependent helicase	*Calidifontibacillus erzurumensis*	466	99	4.00E-157	49.60	499	WP_173731094.1
ATP-dependent helicase	*Romboutsia* sp. 1001713B170131_170501_G6	434	96	3.00E-144	48.78	503	WP_195940152.1
ATP-dependent helicase	*Alteribacillus iranensis*	478	99	7.00E-162	48.68	497	WP_091661275.1

Table S3. Top ten structural neighbors of GajB identified by the Dali server (2)

Protein	Organism	PDB ID	Z-score	RMSD (Å)	Number of superposed residues
UvrD	*Escherichia coli*	3LFU	19.0	4.7	242
PcrA	*Geobacillus stearothermophilus*	1QHG	18.0	4.6	244
AddA	*Bacillus subtilis*	3U44	16.5	4.4	254
Rep	*Escherichia coli*	1UAA	16.3	4.7	242
RecB	*Escherichia coli* K-12	3K70	15.3	4.2	251
AdnAB	*Mycolicibacterium smegmatis*	7SJR	15.3	4.3	240
DNA packaging protein Gp17	*Escherichia virus T4*	2O0H	13.1	2.8	139
DEAH box polypeptide 37 (DHX37)	*Mus musculus*	6O16	12.8	3.4	162
Putative mRNA splicing factor	*Chaetomium thermophilum* var. *thermophilum* DSM 1495	6FAC	12.4	3.4	168
Pre-mRNA-splicing factor, ATP-dependent RNA helicase PRP43	*Chaetomium thermophilum* var. *thermophilum* DSM 1495	5LTA	12.4	4.5	170

Table S4. Top ten structural neighbors of the GajB Nacc subdomain identified by the Dali server (2)

Protein	Organism	PDB ID	Z-score	RMSD (Å)	Number of superposed residues
AddA	*Bacillus subtilis*	3U44	6.2	2.3	70
UvrD	*Escherichia coli*	3LFU	5	2.5	71
PcrA	*Geobacillus stearothermophilus*	4C30	4.4	2.0	67
Transcription regulator AcaB	*Escherichia coli*	6N8A	3.1	3.1	52
Tegument protein UL21	Human alphaherpesvirus 1 strain 17	5ED7	2.7	3.1	55
Exodeoxyribonuclease I	*Escherichia coli* K-12	4JS4	2.6	3.2	60
Adenylate cyclase 9	*Bos taurus*	6R4P	2.6	3.7	49
TNF receptor-associated protein 1	*Danio rerio*	5TVX	2.6	2.7	49
LYR motif-containing protein 4	*Homo sapiens*	6UXE	2.5	3.0	50
Threonine phosphate decarboxylase-like enzyme	*Sulfurospirillum multivorans*	6OUX	2.5	3.6	47

Table S5. Top ten structural neighbors of the GajB Cacc subdomain identified by the Dali server (2)

Protein	Organism	PDB ID	Z-score	RMSD (Å)	Number of superposed residues
Energy-coupling factor transporter	*Levilactobacillus brevis* ATCC 367	4HUQ	5.2	3.4	88
RecB	*Escherichia coli*	6SJE	5.2	3.8	86
RNA polymerase sigma factor RpoD	*Escherichia coli*	6N57	4.9	2.6	65
Rep helicase	*Escherichia coli*	1UAA	4.8	3.3	91
AddB	*Bacillus subtilis*	3U44	4.7	3.2	83
AddA	*Bacillus subtilis*	3U4Q	4.7	3.4	88
40S ribosomal protein eS17	*Plasmodium falciparum* 3D7	3J7A	4.7	3.1	63
PcrA	*Geobacillus stearothermophilus*	2PJR	4.7	3.5	88
ribosomal protein uS15	*Oryctolagus cuniculus*	6YAN	4.5	3.0	60
Polycomb protein SUZ12	*Homo sapiens*	5LS6	4.4	3.4	53

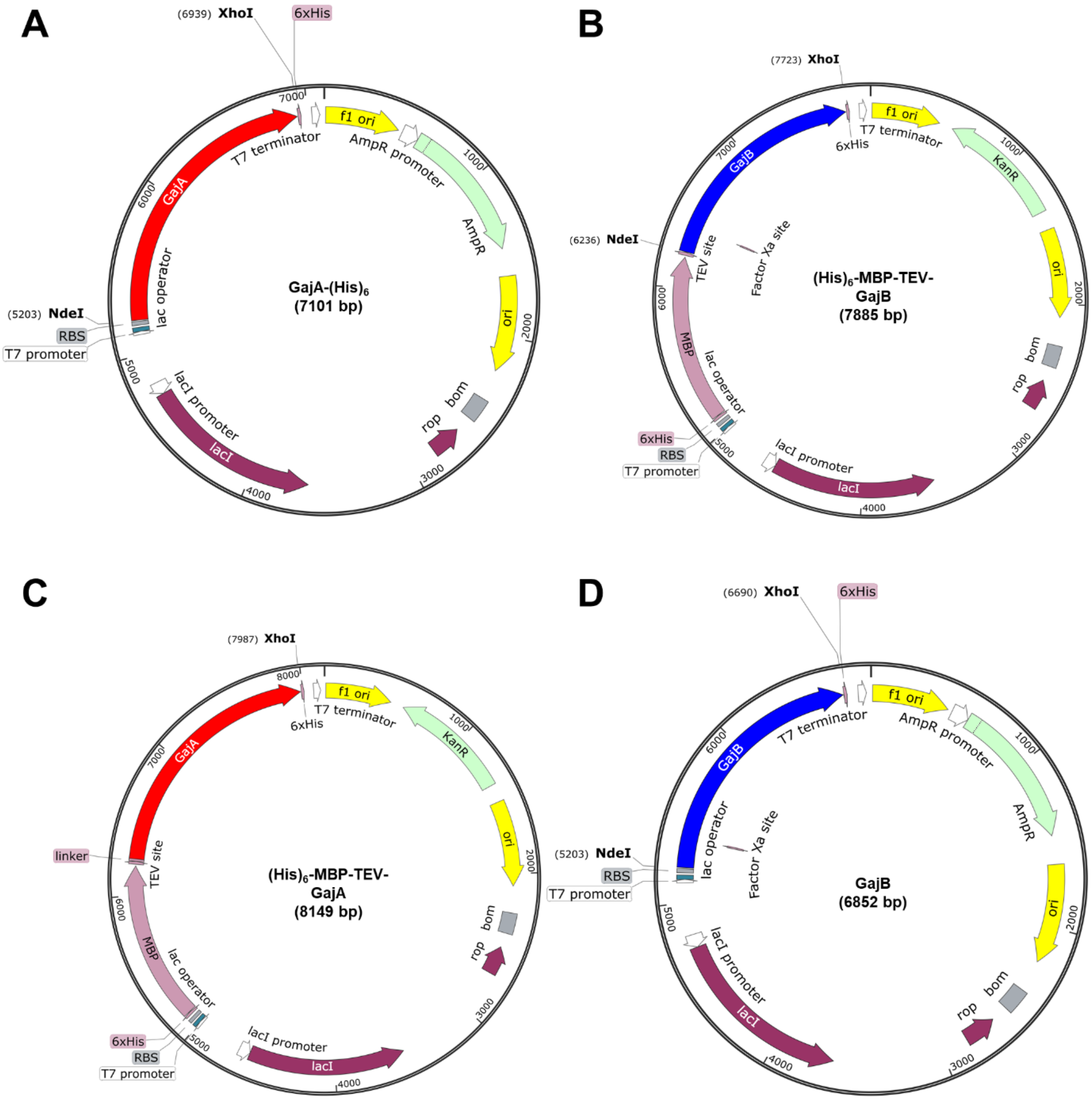

Figure S1. Plasmid maps of expression vectors for Gabija proteins. The maps were generated by using SnapGene software (v.5.3.1).

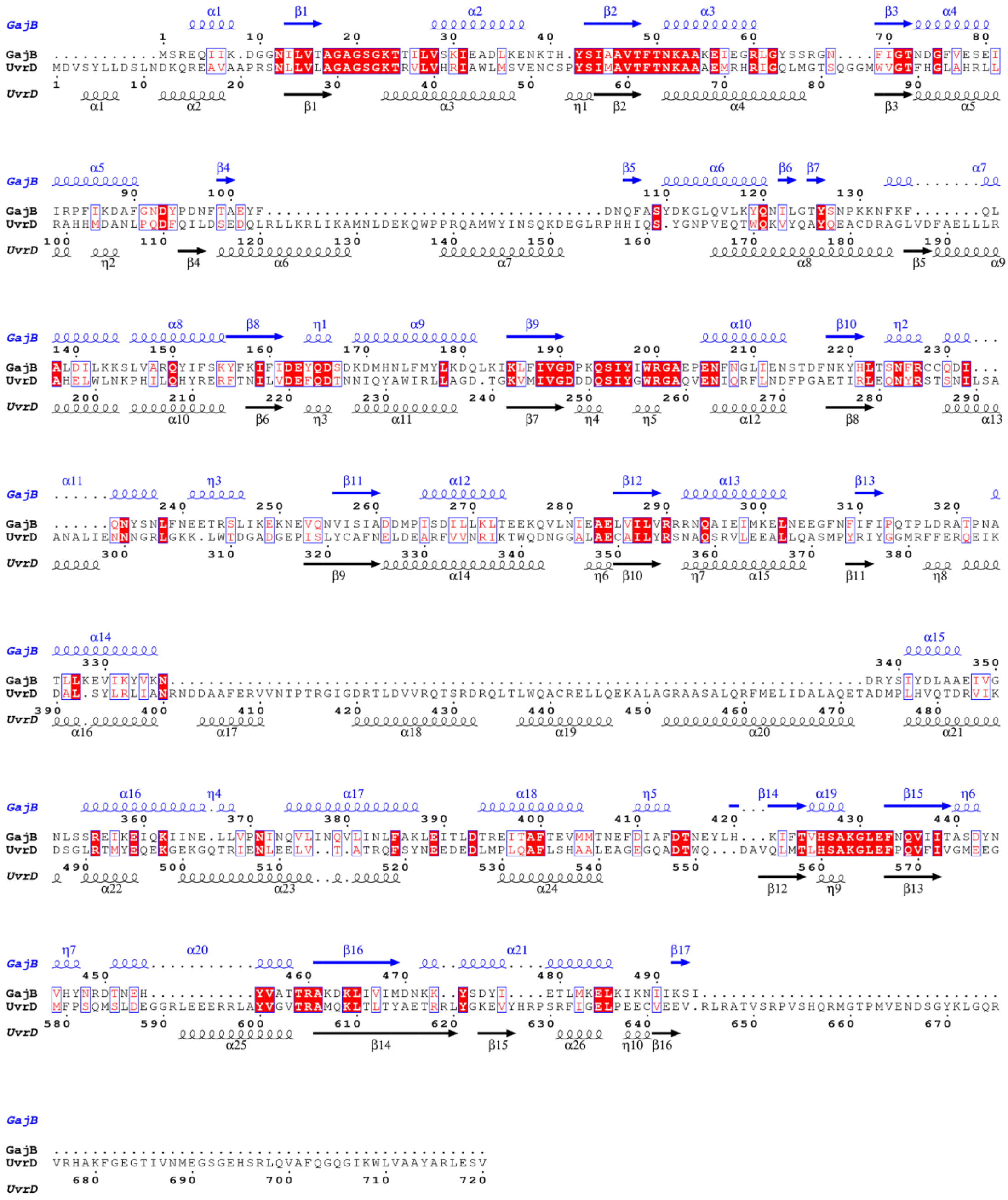

Figure S2. Sequence alignment of GajB and UvrD. White characters in red boxes indicate strict identity. Red characters represent similarity. The secondary structural elements in GajB and UvrD (PDB ID: 3LFU) are shown in blue and black, respectively.

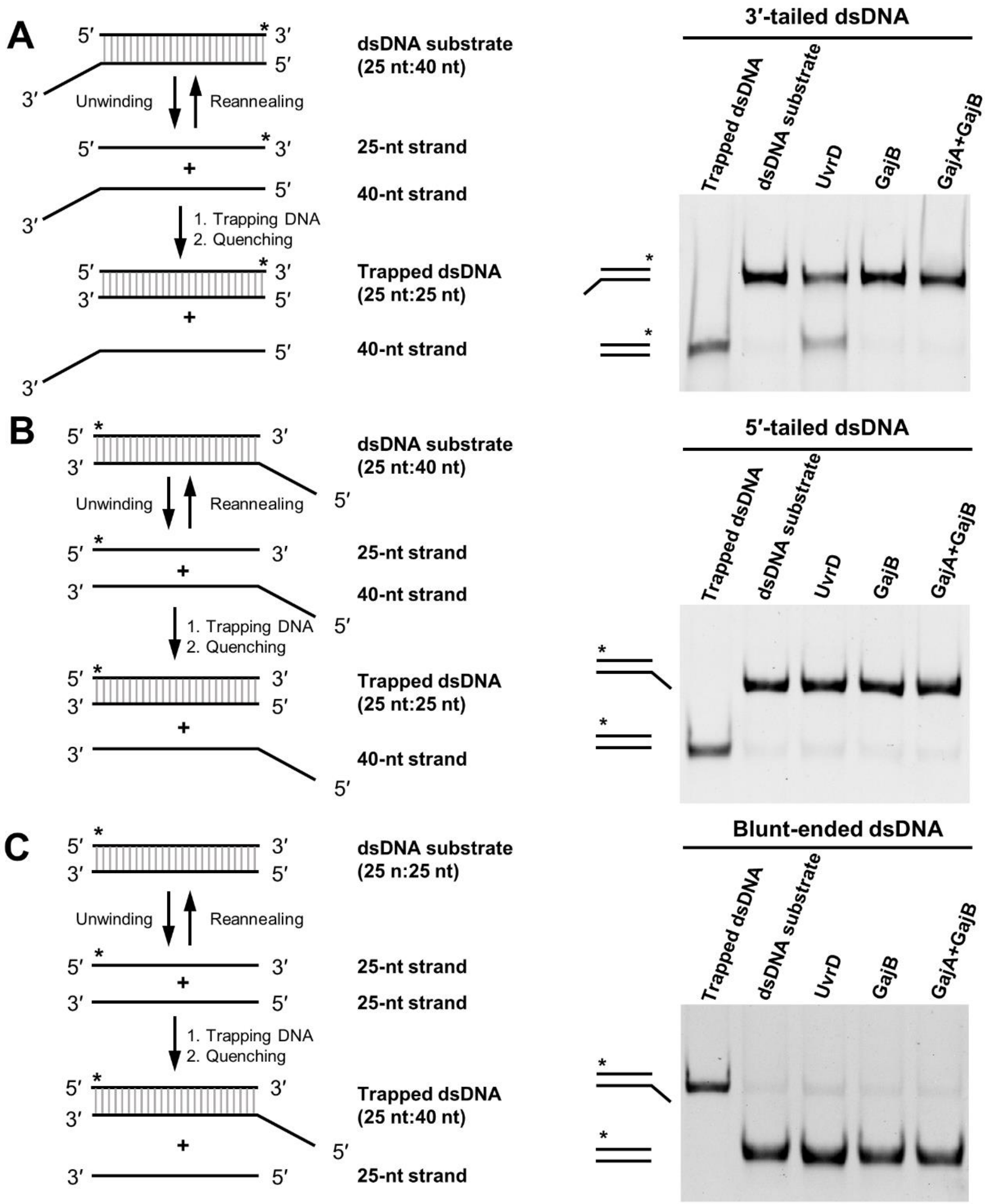

Figure S3. GajB and GajA:GajB complex did not display helicase activity for other DNA substrates. Helicase activity assays were performed with 3´-tailed (A), 5´-tailed (B) and blunt-ended (C) dsDNAs. In the 3´-tailed substrate, fluorescein was labeled at the 3′-end of the 25-nt strand. The DNA substrates were incubated with proteins and analyzed by PAGE. Experimental schemes for the assays are also shown. Asterisks indicate fluorescein labels. Uncropped gel images are shown in Fig. S8.

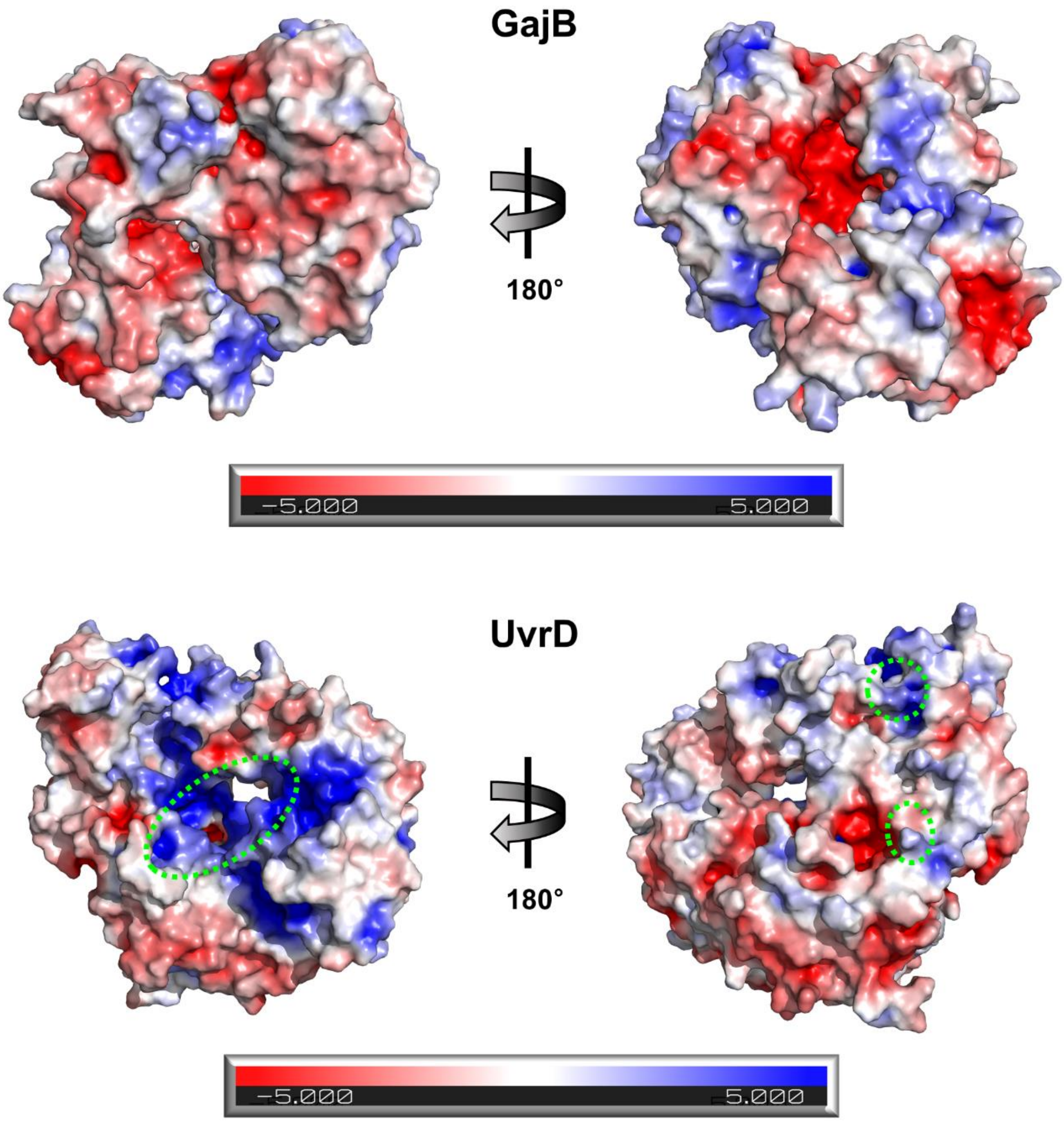

Figure S4. Electrostatic potential surfaces of GajB and UvrD, PyMOL software (the PyMOL Molecular Graphics System, Version 2.0 Schrödinger, LLC) was used with the Adaptive Poisson-Boltzmann Solver plugin to generate the surfaces (red = −5.0 kT, blue = +5.0 kT). The DNA binding interface of UvrD is also indicated. Orientations of the structures shown on left are identical to those in Fig. 2A.

A

Protein	Measured average molar mass (kDa)	Theoretical molecular weight (kDa)
BSA	63.9	66.4
Aldolase	153.0	158
Thyroglobulin	652.8	669

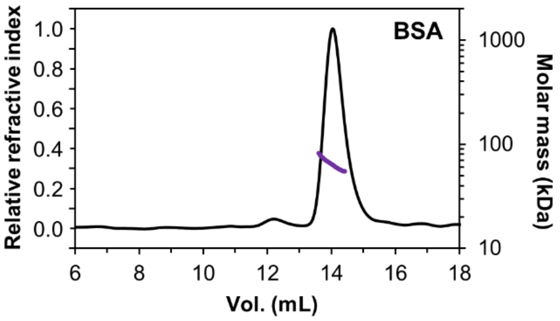

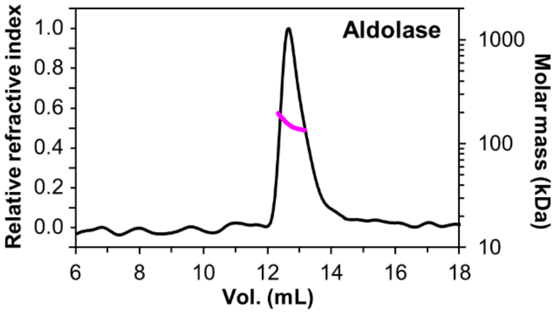

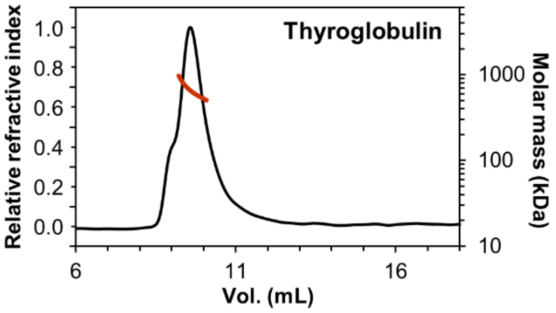

B

No.	Protein	Molecular weight (kDa)	Elution volume (mL)
1	Ferritin	440	11.55
2	Aldolase	158	13.65
3	Conalbumin	75	14.88
4	Carbonic anhydrase	29	16.76
5	Ribonuclease A	13.7	17.95
6	Aprotinin	6.5	19.21

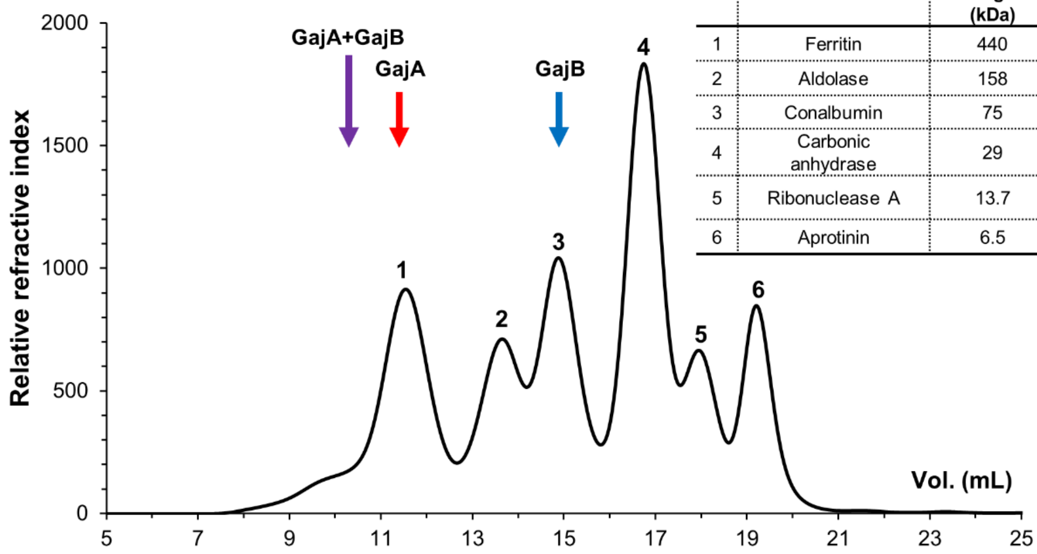

Figure S5. SEC-MALS and analytical SEC of standard proteins. (A) SEC-MALS analyses of standard proteins. The measured average molar masses corresponding the SEC peaks are shown with theoretical molecular weights of the standard proteins. (B) Analytical SEC experiment of standard proteins. The analytical SEC chromatogram of the standard proteins is shown with their theoretical molecular weights. The elution volumes of GajA, GajB and GajA:GajB complex are also indicated.

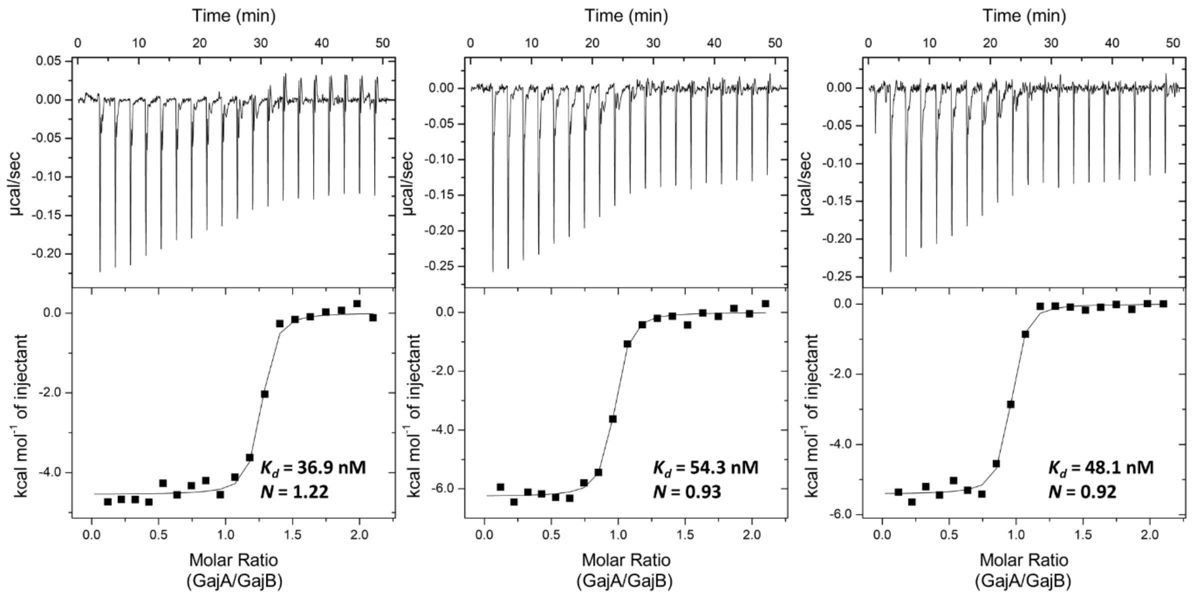

Figure S6. ITC traces for GajA binding to GajB. Three independent experiments were performed to calculate average values for the dissociation constant (*Kd*) and molar binding ratio (*N*). The data from the third ITC run are presented as representative in Fig. 5G. The *Kd* and *N* values for each measurement are also shown.

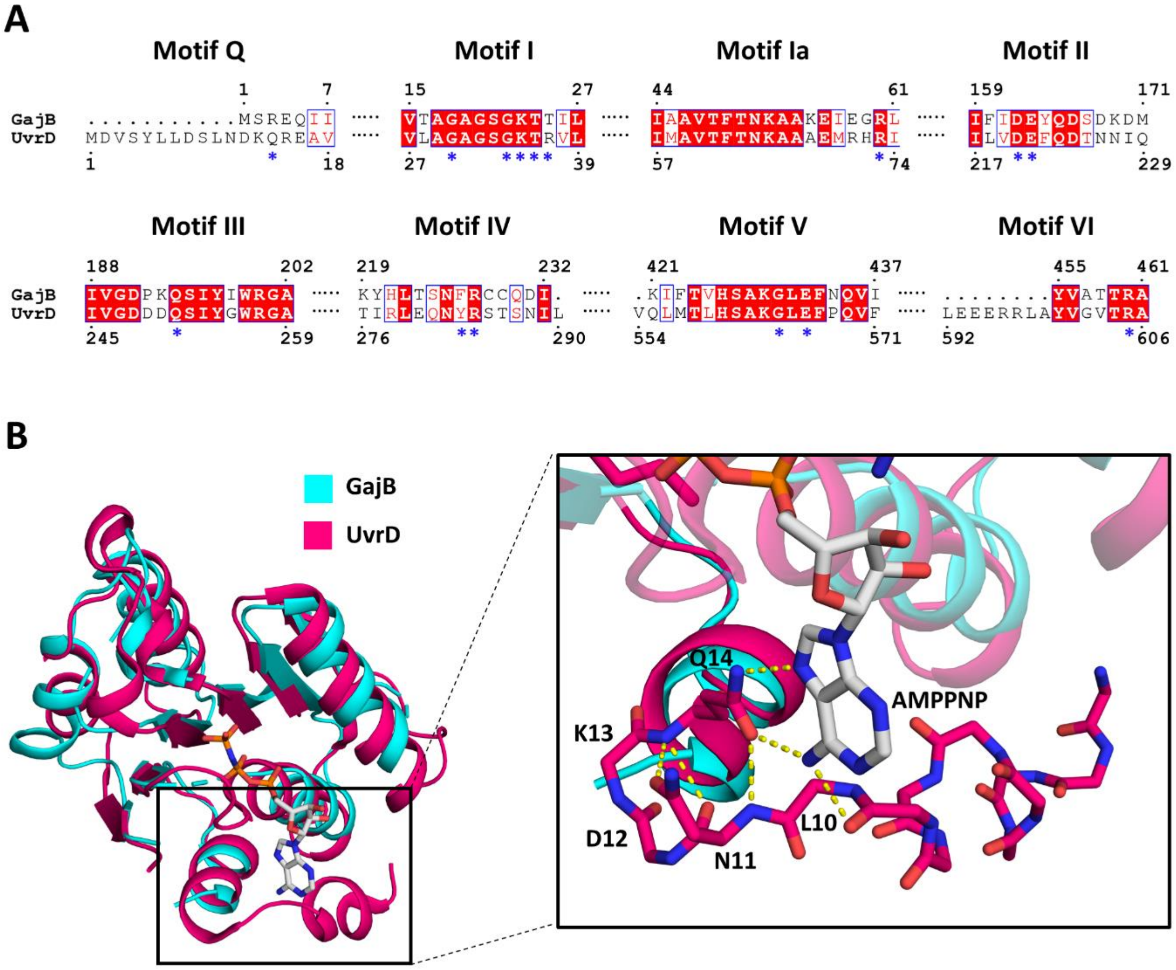

Figure S7. Comparison of the ATP-binding region between GajB and UvrD. (A) Structure-guided sequence alignment of GajB and UvrD in eight helicase motifs required for ATP hydrolysis in SF1 helicases. GajB lacks several N-terminal residues in Motif Q. Blue asterisks indicate ATP-binding residues in UvrD. (B) Structural comparison of the N-terminal region between GajB (cyan) and AMPPNP-bound UvrD (PDB ID: 2IS4; red). Superposition of the Ncore subdomain in GajB with the 1A subdomain in UvrD reveals the absence of N-terminal residues in GajB, which, in the UvrD structure, interact with the base ring of AMPPNP.

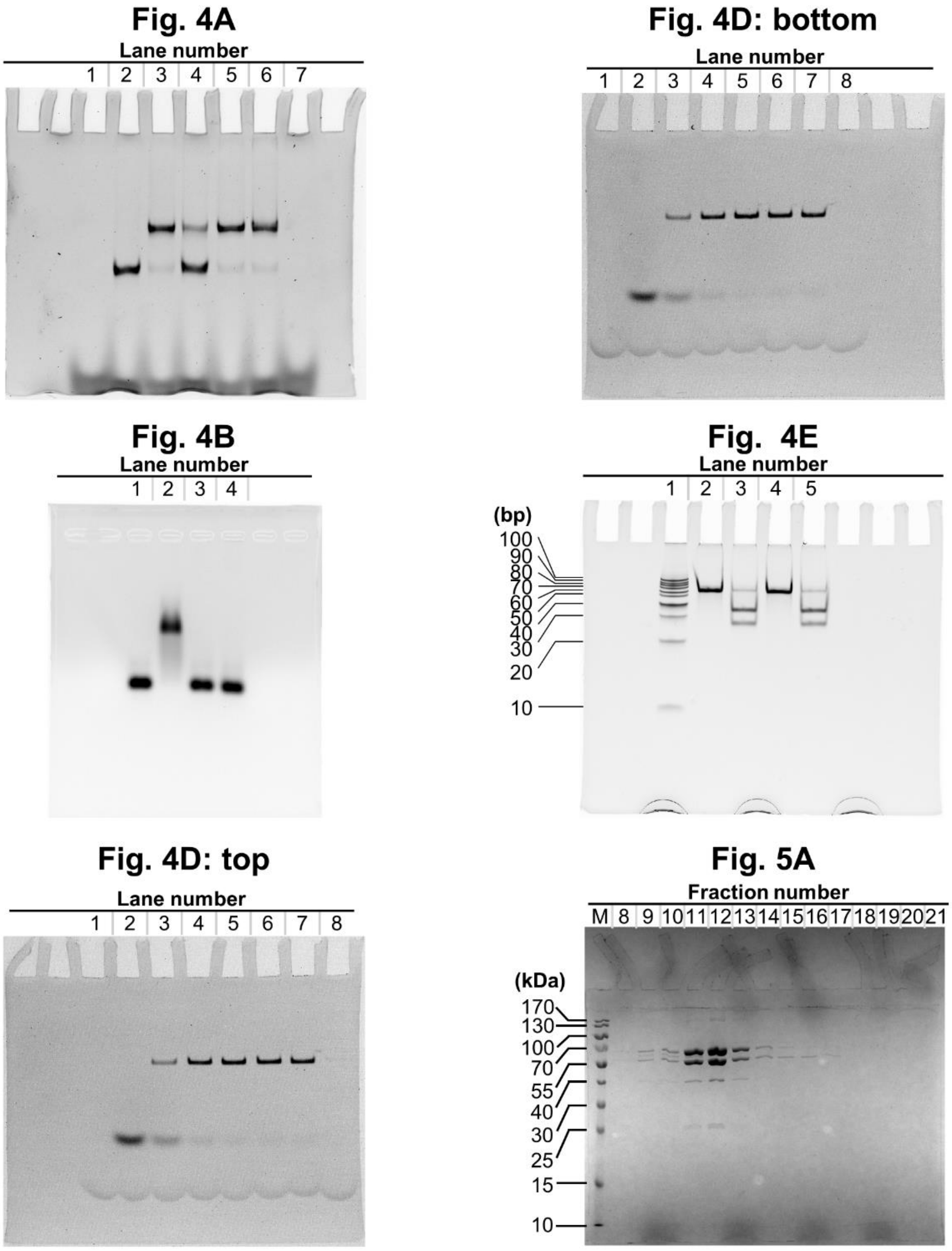

Figure S8. Uncropped gel images.

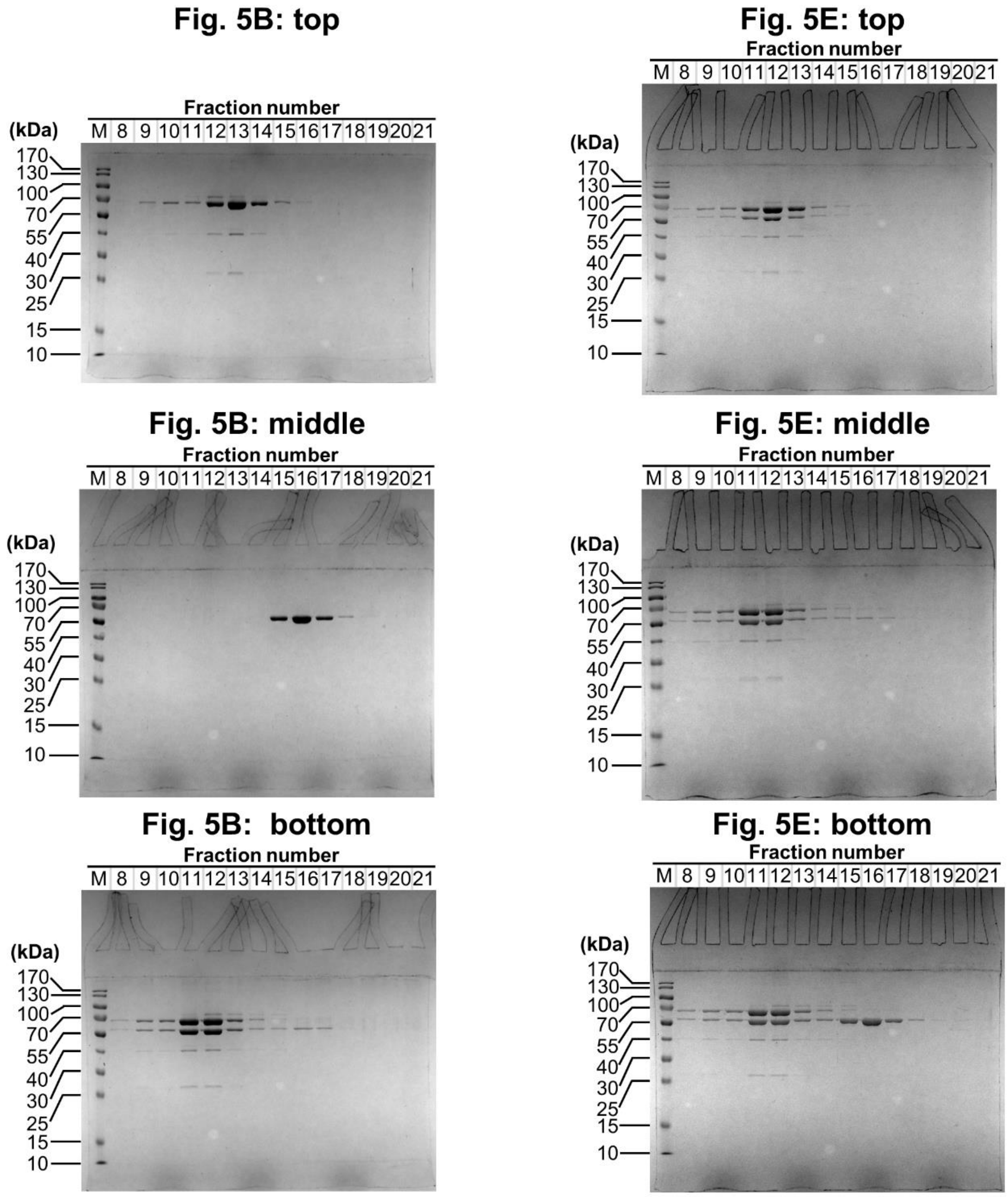

Figure S8. Uncropped gel images (continued).

Fig. S3A

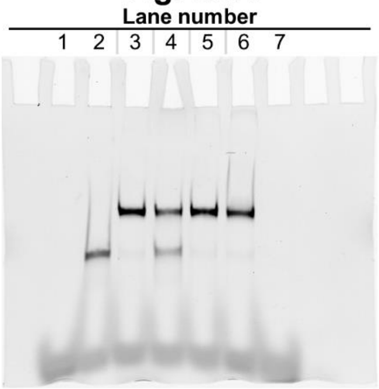

Fig. S3B

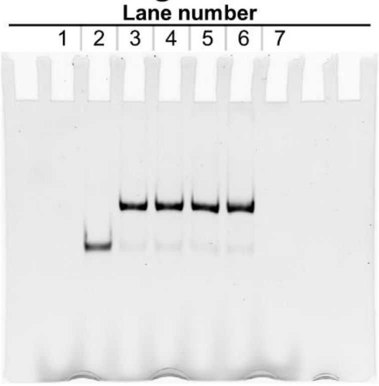

Fig. S3C

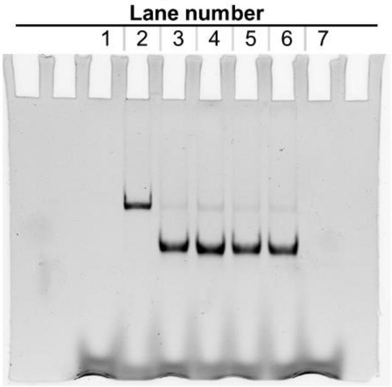

Figure S8. Uncropped gel images (continued).

Supplementary References

1. Altschul, S.F., Gish, W., Miller, W., Myers, E.W. and Lipman, D.J. (1990) Basic local alignment search tool. J Mol Biol, 215, 403-410.
2. Holm, L., Kaariainen, S., Rosenstrom, P. and Schenkel, A. (2008) Searching protein structure databases with DaliLite v.3. Bioinformatics, 24, 2780-2781.
